# A generalization of a theorem of Bor

**DOI:** 10.1186/s13660-017-1455-3

**Published:** 2017-08-02

**Authors:** Hikmet Seyhan Özarslan, Bağdagül Kartal

**Affiliations:** Department of Mathematics, Erciyes University, Kayseri, 38039 Turkey

**Keywords:** 26D15, 40D15, 40F05, 40G99, matrix transformations, almost increasing sequences, quasi-monotone sequences, Hölder inequality, Minkowski inequality

## Abstract

In this paper, a general theorem concerning absolute matrix summability is established by applying the concepts of almost increasing and *δ*-quasi-monotone sequences.

## Introduction

A positive sequence $(y_{n})$ is said to be almost increasing if there is a positive increasing sequence $(u_{n})$ and two positive constants K and M such that $Ku_{n} \leq y_{n} \leq Mu_{n}$ (see [1]). A sequence $(c_{n})$ is said to be *δ*-quasi-monotone, if $c_{n}\rightarrow 0$, $c_{n}>0$ ultimately and $\Delta c_{n}\geq-\delta_{n}$, where $\Delta c_{n}=c_{n}-c_{n+1}$ and $\delta=(\delta_{n})$ is a sequence of positive numbers (see [2]). Let $\sum a_{n}$ be a given infinite series with partial sums $(s_{n})$. Let $T=(t_{nv})$ be a normal matrix, *i.e.*, a lower triangular matrix of nonzero diagonal entries. At that time *T* describes the sequence-to-sequence transformation, mapping the sequence $s=(s_{n})$ to $Ts=(T_{n}(s))$, where

1 $$ T_{n}(s)=\sum_{v=0}^{n}t_{nv}s_{v}, \quad n=0,1,\ldots $$

Let $(\varphi_{n})$ be any sequence of positive real numbers. The series $\sum{a_{n}}$ is said to be summable ${\varphi}-|T,p_{n}|_{k}$, $k\geq 1$, if (see [3])

2 $$ \sum_{n=1}^{\infty} \varphi_{n}^{k-1}\big| \bar{\Delta}T_{n}(s)\big|^{k}< \infty, $$

where

$$ \bar{\Delta}T_{n}(s)=T_{n}(s)-T_{n-1}(s). $$

If we take $\varphi_{n}=\frac{P_{n}}{p_{n}}$, then ${\varphi }-|T,p_{n}|_{k}$ summability reduces to $|T,p_{n}|_{k}$ summability (see [4]). If we set $\varphi_{n}=n$ for all *n*, $\varphi- \vert T,p_{n} \vert _{k}$ summability is the same as $\vert T \vert _{k}$ summability (see [5]). Also, if we take $\varphi_{n}=\frac{P_{n}}{p_{n}}$ and $t_{nv}=\frac {p_{v}}{P_{n}}$, then we get $|\bar{N},p_{n}|_{k}$ summability (see [6]).

## Known result

In [7, 8], Bor has established the following theorem dealing with $\vert \bar{N},p_{n} \vert _{k}$ summability factors of infinite series.

### Theorem 2.1

*Let* $(Y_{n})$ *be an almost increasing sequence such that* $|\Delta{Y_{n}}|=O(Y_{n}/n)$ *and* $\lambda_{n}\rightarrow0$ *as* $n\rightarrow\infty$. *Assume that there is a sequence of numbers* $(B_{n})$ *such that it is* *δ*-*quasi*-*monotone with* $\sum nY_{n}\delta_{n}<\infty$, $\sum B_{n}Y_{n}$ *is convergent and* $|\Delta\lambda_{n}|\leq|B_{n}|$ *for all* *n*. *If*

3 $$\begin{aligned}& \sum_{n=1}^{m} \frac{1}{n}|\lambda_{n}|= O(1)\quad \textit{as }m\rightarrow\infty, \end{aligned}$$

4 $$\begin{aligned}& \sum_{n=1}^{m} \frac{1}{n}|z_{n}|^{k}= O(Y_{m})\quad \textit{as }m \rightarrow\infty, \end{aligned}$$

*and*

5 $$ \sum_{n=1}^{m} \frac{p_{n}}{P_{n}}|z_{n}|^{k}= O(Y_{m})\quad \textit{as }m \rightarrow\infty, $$

*where* $(z_{n})$ *is the* *nth* $(C,1)$ *mean of the sequence* $(n a_{n})$, *then the series* $\sum a_{n}\lambda_{n}$ *is summable* $|\bar{N},p_{n}|_{k}$, $k\geq1$.

## Main result

The purpose of this paper is to generalize Theorem 2.1 to the $\varphi - \vert T,p_{n} \vert _{k}$ summability. Before giving main theorem, let us introduce some well-known notations. Let $T= (t_{nv} )$ be a normal matrix. Lower semimatrices $\bar{T}=(\bar{t}_{nv})$ and $\hat{T}=(\hat{t}_{nv})$ are defined as follows:

6 $$ \bar{t}_{nv}=\sum_{i=v}^{n}t_{ni}, \quad n,v=0,1,\ldots $$

and

7 $$ \hat{t}_{00}=\bar{t}_{00}=t_{00} , \qquad \hat{t}_{nv}=\bar {t}_{nv}-\bar{t}_{n-1,v},\quad n=1,2,\ldots $$

Here, *T̄* and *T̂* are the well-known matrices of series-to-sequence and series-to-series transformations, respectively. Then we write

8 $$ T_{n} (s ) = \sum_{v=0}^{n}t_{nv}s_{v}= \sum_{v=0}^{n}\bar {t}_{nv}a_{v} $$

and

9 $$ \bar{\Delta}T_{n} (s ) = \sum _{v=0}^{n}\hat{t}_{nv}a_{v}. $$

By taking the definition of general absolute matrix summability, we established the following theorem.

### Theorem 3.1

*Let* $T=(t_{nv})$ *be a positive normal matrix such that*

10 $$\begin{aligned}& \bar{t}_{n0}=1, \quad n=0,1,\ldots, \end{aligned}$$

11 $$\begin{aligned}& t_{n-1,v} \geq t_{nv}, \quad \textit{for }n \geq v+1, \end{aligned}$$

12 $$\begin{aligned}& t_{nn}=O \biggl( \frac{p_{n}}{P_{n}} \biggr), \end{aligned}$$

*and* $(\frac{\varphi_{n}p_{n}}{P_{n}} )$ *be a non*-*increasing sequence*. *If all conditions of Theorem* 2.1 *with conditions* (4) *and* (5) *are replaced by*

13 $$ \sum_{n=1}^{m} \varphi_{n}^{k-1} \biggl( \frac{p_{n}}{P_{n}} \biggr) ^{k-1}\frac{1}{n}|z_{n}|^{k} = O(Y_{m}) \quad\textit{as } {m\rightarrow\infty} $$

*and*

14 $$ \sum_{n=1}^{m} \varphi_{n}^{k-1} \biggl( \frac{p_{n}}{P_{n}} \biggr) ^{k}|z_{n}|^{k} = O(Y_{m}) \quad\textit{as } {m \rightarrow\infty}, $$

*then the series* $\sum a_{n} \lambda_{n}$ *is* ${\varphi}-|T,p_{n}|_{k}$ *summable*, $k\geq1$.

We need the following lemmas for the proof of Theorem 3.1.

### Lemma 3.2

[7]

*Let* $(Y_{n})$ *be an almost increasing sequence and* $\lambda_{n}\rightarrow0$ *as* $n\rightarrow\infty$. *If* $(B_{n})$ *is* *δ*-*quasi*-*monotone with* $\sum B_{n}Y_{n}$ *is convergent and* $|\Delta\lambda_{n}|\leq|B_{n}|$ *for all* *n*, *then we have*

15 $$ \vert \lambda_{n} \vert Y_{n}=O (1 ) \quad \textit{as }n\rightarrow\infty. $$

### Lemma 3.3

[8]

*Let* $(Y_{n})$ *be an almost increasing sequence such that* $n| {\Delta Y_{n}}|=O (Y_{n} )$. *If* $(B_{n})$ *is* *δ*-*quasi monotone with* $\sum nY_{n}\delta_{n}<\infty$, *and* $\sum B_{n}Y_{n}$ *is convergent*, *then*

16 $$\begin{aligned}& n B_{n} Y_{n}=O (1 ) \quad \textit{as }n\rightarrow \infty, \end{aligned}$$

17 $$\begin{aligned}& \sum_{n=1}^{\infty}nY_{n}| \Delta B_{n}|< \infty. \end{aligned}$$

## Proof of Theorem 3.1

Let $(I_{n})$ indicate the *T*-transform of the series $\sum a_{n}\lambda_{n}$. Then we obtain

18 $$ \bar{\Delta}I_{n} = \sum _{v=0}^{n}\hat{t}_{nv}a_{v} \lambda_{v}=\sum_{v=1}^{n} \frac{\hat{t}_{nv}\lambda_{v}}{v} v a_{v} $$

by means of (8) and (9).

Using Abel’s formula for (18), we obtain

$$\begin{aligned} \bar{\Delta}I_{n} ={} & \sum_{v=1}^{n-1} \Delta_{v} \biggl( \frac{\hat{t}_{nv}\lambda _{v}}{v} \biggr) \sum _{r=1}^{v} ra_{r}+\frac{\hat{t}_{nn}\lambda _{n}}{n}\sum _{r=1}^{n} ra_{r} \\ ={} & \sum_{v=1}^{n-1}\frac{v+1}{v} \Delta_{v} (\hat{t}_{nv} )\lambda_{v}z_{v}+ \sum_{v=1}^{n-1}\frac{v+1}{v} \hat{t}_{n,v+1}\Delta \lambda_{v}z_{v} \\ &+\sum_{v=1}^{n-1}\hat{t}_{n,v+1} \lambda _{v+1}\frac{z_{v}}{v}+\frac{n+1}{n}{t}_{nn} \lambda_{n}z_{n} \\ = {}& I_{n,1}+I_{n,2}+I_{n,3}+I_{n,4}. \end{aligned}$$

For the proof of Theorem 3.1, it suffices to prove that

$$ \sum_{n=1}^{\infty}\varphi_{n}^{k-1} \vert I_{n,r} \vert ^{k}< \infty $$

for $r=1,2,3,4$.

By Hölder’s inequality, we have

$$\begin{aligned} \sum_{n=2}^{m+1}\varphi_{n}^{k-1}| I_{n,1}|^{k} & = O(1) \sum_{n=2}^{m+1} \varphi_{n}^{k-1} \Biggl(\sum_{v=1}^{n-1} \bigl\vert \Delta_{v}(\hat{t}_{nv}) \bigr\vert \vert \lambda_{v} \vert \vert z_{v} \vert \Biggr)^{k} \\ &= O(1) \sum_{n=2}^{m+1} \varphi_{n}^{k-1} \Biggl(\sum_{v=1}^{n-1} \bigl\vert \Delta_{v}(\hat{t}_{nv}) \bigr\vert \vert \lambda_{v} \vert ^{k} \vert z_{{v}} \vert ^{k} \Biggr) \Biggl(\sum_{v=1}^{n-1}\big| \Delta_{v}(\hat{t}_{nv})\big| \Biggr) ^{k-1}. \end{aligned}$$

By (6) and (7), we have

19 $$ \begin{aligned}[b]\Delta_{v}(\hat{t}_{nv})&= \hat{t}_{nv}-\hat{t}_{n,v+1} \\ &=\bar{t}_{nv}-\bar{t}_{n-1,v}-\bar{t}_{n,v+1}+\bar {t}_{n-1,v+1} \\ &= {t}_{nv}-{t}_{n-1,v}. \end{aligned}$$

Thus using (6), (10) and (11)

$$ \sum_{v=1}^{n-1} \big|\Delta_{v}( \hat{t}_{nv})\big|=\sum_{v=1}^{n-1}(t_{n-1,v}-t_{nv}) \leq t_{nn}. $$

Hence, we get

$$ \sum_{n=2}^{m+1}\varphi_{n}^{k-1}|I_{n,1}|^{k} = O(1)\sum_{n=2}^{m+1} \varphi_{n}^{k-1} {t}_{nn}^{k-1} \Biggl(\sum _{v=1}^{n-1} \bigl\vert \Delta_{v}( \hat{t}_{nv}) \bigr\vert \vert \lambda_{v} \vert ^{k} \vert z_{{v}} \vert ^{k} \Biggr) $$

by using (12)

$$\begin{aligned} \sum_{n=2}^{m+1}\varphi_{n}^{k-1}| I_{n,1}|^{k} & =O(1)\sum_{n=2}^{m+1} \biggl( \frac{\varphi _{n}{p_{n}}}{P_{n}} \biggr) ^{k-1} \Biggl(\sum _{v=1}^{n-1} \bigl\vert \Delta_{v}( \hat{t}_{nv}) \bigr\vert \vert \lambda_{v} \vert ^{k} \vert z_{{v}} \vert ^{k} \Biggr) \\ &= O(1)\sum_{v=1}^{m} | \lambda_{v} |^{k}| z_{v} |^{k} \sum _{n=v+1}^{m+1} \biggl( \frac{\varphi_{n}{p_{n}}}{P_{n}} \biggr) ^{k-1} \big|\Delta_{v}(\hat{t}_{nv})\big| \\ &= O(1)\sum_{v=1}^{m} \biggl( \frac{\varphi_{v}{p_{v}}}{P_{v}} \biggr) ^{k-1} |\lambda_{v} |^{k}| z_{v} |^{k} \sum_{n=v+1}^{m+1} \big|\Delta_{v}(\hat{t}_{nv})\big|. \end{aligned}$$

Now, using (11) and (19), we obtain

$$ \sum_{n=v+1}^{m+1} \big|\Delta_{v}( \hat{t}_{nv})\big|=\sum_{n=v+1}^{m+1}(t_{n-1,v}-t_{nv}) \leq t_{vv}. $$

Thus, by using Abel’s formula, we obtain

$$\begin{aligned} \sum_{n=2}^{m+1}\varphi_{n}^{k-1}| I_{n,1}|^{k}&= O(1)\sum_{v=1}^{m} \biggl( \frac{\varphi _{v}{p_{v}}}{P_{v}} \biggr) ^{k-1} |\lambda_{v} |^{k-1}|\lambda_{v} || z_{v} |^{k} t_{vv} \\ &= O(1)\sum_{v=1}^{m}\varphi_{v}^{k-1} \biggl( \frac{p_{v}}{P_{v}} \biggr) ^{k} \vert \lambda_{v} \vert \vert z_{v} \vert ^{k} \\ & = O(1) \sum_{v=1}^{m-1}\Delta| \lambda_{v} |\sum_{r=1}^{v} \varphi _{r}^{k-1} \biggl( \frac{p_{r}}{P_{r}} \biggr) ^{k}| z_{r} |^{k}+O(1)| \lambda_{m} | \sum_{v=1}^{m} \varphi_{v}^{k-1} \biggl( \frac {p_{v}}{P_{v}} \biggr) ^{k}|z_{v}|^{k} \\ &= O(1)\sum_{v=1}^{m-1}| \Delta \lambda_{v} | Y_{v} + O(1)| \lambda_{m} | Y_{m} \\ &= O(1)\sum_{v=1}^{m-1}| B_{v}| Y_{v}+O(1)|\lambda_{m}|Y_{m} \\ & = O(1) \quad \text{as }m\rightarrow\infty, \end{aligned}$$

in view of (14) and (15).

Again, using Hölder’s inequality, we have

$$\begin{aligned} \sum_{n=2}^{m+1}\varphi_{n}^{k-1}| I_{n,2}|^{k} ={} & O(1) \sum_{n=2}^{m+1} \varphi_{n}^{k-1} \Biggl(\sum_{v=1}^{n-1}| \hat {t}_{n,v+1}||\Delta\lambda_{v}||z_{v}| \Biggr)^{k} \\ ={} & O(1) \sum_{n=2}^{m+1} \varphi_{n}^{k-1} \Biggl(\sum_{v=1}^{n-1}| \hat {t}_{n,v+1}||B_{v}||z_{v}|^{k} \Biggr) \\ &\times \Biggl(\sum_{v=1}^{n-1}|\hat {t}_{n,v+1}||B_{v}| \Biggr)^{k-1}. \end{aligned}$$

By means of (6), (7) and (11), we have

$$\begin{aligned} \hat{t}_{n,v+1}&= \bar{t}_{n,v+1}-\bar{t}_{n-1,v+1} \\ &= \sum_{i=v+1}^{n} t_{ni}-\sum _{i=v+1}^{n-1} t_{n-1,i} \\ &= t_{nn}+\sum_{i=v+1}^{n-1} ( t_{ni}- t_{n-1,i} ) \\ &\leq t_{nn}. \end{aligned}$$

In this way, we have

$$\begin{aligned} \sum_{n=2}^{m+1}\varphi_{n}^{k-1}| I_{n,2}|^{k}& = O(1) \sum_{n=2}^{m+1} \varphi_{n}^{k-1}{t}_{nn}^{k-1} \Biggl( \sum _{v=1}^{n-1}|\hat{t}_{n,v+1}||B_{v}||z_{v}|^{k} \Biggr) \\ & = O(1) \sum_{n=2}^{m+1} \biggl( \frac{\varphi_{n}{p_{n}}}{P_{n}} \biggr) ^{k-1} \Biggl( \sum _{v=1}^{n-1}|\hat{t}_{n,v+1}||B_{v}||z_{v}|^{k} \Biggr) \\ & = O(1)\sum_{v=1}^{m}|B_{v}||z_{v}|^{k} \sum_{n=v+1}^{m+1} \biggl( \frac{\varphi_{n}{p_{n}}}{P_{n}} \biggr) ^{k-1}|\hat {t}_{n,v+1}| \\ &= O(1)\sum_{v=1}^{m} \biggl( \frac{\varphi _{v}{p_{v}}}{P_{v}} \biggr) ^{k-1}|B_{v}||z_{v}|^{k} \sum_{n=v+1}^{m+1}|\hat {t}_{n,v+1}|. \end{aligned}$$

By (6), (7), (10) and (11), we obtain

$$ |\hat{t}_{n,v+1}|=\sum_{i=0}^{v} (t_{n-1,i}-t_{ni}). $$

Thus, using (6) and (10), we have

$$ \sum_{n=v+1}^{m+1} |\hat{t}_{n,v+1}|= \sum_{n=v+1}^{m+1} \sum _{i=0}^{v} (t_{n-1,i}-t_{ni})\leq1, $$

then we get

$$\begin{aligned} \sum_{n=2}^{m+1}\varphi_{n}^{k-1}| I_{n,2}|^{k} ={} & O(1)\sum_{v=1}^{m} \varphi_{v}^{k-1} \biggl( \frac{p_{v}}{P_{v}} \biggr) ^{k-1} v |B_{v}|\frac{1}{v}|z_{v}|^{k} \\ ={} & O(1)\sum_{v=1}^{m-1} \Delta\bigl(v|B_{v}|\bigr)\sum_{r=1}^{v} \varphi_{r}^{k-1} \biggl( \frac {p_{r}}{P_{r}} \biggr) ^{k-1} \frac{1}{r} | z_{r} |^{k} \\ &+ O(1)m|B_{m}| \sum_{v=1}^{m} \varphi_{v}^{k-1} \biggl( \frac {p_{v}}{P_{v}} \biggr) ^{k-1} \frac{1}{v}| z_{v} |^{k} \\ ={} & O(1)\sum_{v=1}^{m-1}v|\Delta B_{v}|Y_{v}+O(1)\sum_{v=1}^{m-1}|B_{v}|Y_{v}+O(1)m|B_{m}|Y_{m} \\ ={} & O(1)\quad \text{as }m\rightarrow\infty, \end{aligned}$$

in view of (13), (16) and (17).

Also, we have

$$\begin{aligned} \sum_{n=2}^{m+1}\varphi_{n}^{k-1}|I_{n,3}|^{k} \leq{}& \sum_{n=2}^{m+1}\varphi_{n}^{k-1} \Biggl(\sum_{v=1}^{n-1}|\hat {t}_{n,v+1}||\lambda_{v+1}|\frac{|z_{v}|}{v} \Biggr)^{k} \\ \leq{}& \sum_{n=2}^{m+1}\varphi_{n}^{k-1} \Biggl(\sum_{v=1}^{n-1}|\hat {t}_{n,v+1}||\lambda_{v+1}|\frac{|z_{v}|^{k}}{v} \Biggr) \Biggl(\sum _{v=1}^{n-1}|\hat{t}_{n,v+1}| \frac{|\lambda_{v+1}|}{v} \Biggr)^{k-1} \\ \leq{}& \sum_{n=2}^{m+1} \varphi_{n}^{k-1} t_{nn}^{k-1} \Biggl(\sum _{v=1}^{n-1}|\hat{t}_{n,v+1}|| \lambda_{v+1}|\frac{|z_{v}|^{k}}{v} \Biggr) \Biggl(\sum _{v=1}^{n-1} \frac{|\lambda_{v+1}|}{v} \Biggr)^{k-1} \\ ={} & O(1)\sum_{n=2}^{m+1} \varphi_{n}^{k-1} \biggl(\frac{p_{n}}{P_{n}} \biggr) ^{k-1} \Biggl(\sum_{v=1}^{n-1}| \hat{t}_{n,v+1}||\lambda_{v+1}|\frac {|z_{v}|^{k}}{v} \Biggr) \\ ={} & O(1)\sum_{n=2}^{m+1} \biggl( \frac{\varphi_{n}{p_{n}}}{P_{n}} \biggr) ^{k-1} \Biggl(\sum _{v=1}^{n-1}| \hat{t}_{n,v+1}|| \lambda_{v+1}| \frac {|z_{v}|^{k}}{v} \Biggr) \\ ={} & O(1)\sum_{v=1}^{m}| \lambda_{v+1}|\frac {|z_{v}|}{v}^{k}\sum _{n=v+1}^{m+1} \biggl( \frac{\varphi _{n}{p_{n}}}{P_{n}} \biggr) ^{k-1}|\hat{t}_{n,v+1}| \\ ={} & O(1)\sum_{v=1}^{m} \biggl( \frac{\varphi_{v}{p_{v}}}{P_{v}} \biggr) ^{k-1}|\lambda_{v+1}| \frac{|z_{v}|}{v}^{k}\sum_{n=v+1}^{m+1}| \hat {t}_{n,v+1}| \\ ={} & O(1)\sum_{v=1}^{m} \varphi_{v}^{k-1} \biggl( \frac {{p_{v}}}{P_{v}} \biggr) ^{k-1}|\lambda_{v+1}|\frac{|z_{v}|}{v}^{k} \\ ={}& O(1) \sum_{v=1}^{m-1}|\Delta \lambda_{v+1} |\sum_{r=1}^{v} \varphi _{r}^{k-1} \biggl( \frac{p_{r}}{P_{r}} \biggr) ^{k-1} \frac{1}{r}| z_{r} |^{k} \\ &+ O(1)| \lambda_{m+1} | \sum_{v=1}^{m} \varphi _{v}^{k-1} \biggl( \frac{p_{v}}{P_{v}} \biggr)^{k-1} \frac{1}{v}|z_{v} |^{k} \\ ={} & O(1)\sum_{v=1}^{m-1}|B_{v+1}| Y_{v+1}+O(1) |\lambda_{m+1} | Y_{m+1} \\ ={} & O(1)\quad \text{as }m\rightarrow\infty, \end{aligned}$$

in view of (3), (12), (13) and (15).

Finally, as in $I_{n,1}$, we have

$$\begin{aligned} \sum_{n=1}^{m}\varphi_{n}^{k-1}| I_{n,4}|^{k} & = O(1)\sum_{n=1}^{m} \varphi_{n}^{k-1} {t}_{nn}^{k} | \lambda_{n} |^{k} |z_{n}|^{k} \\ & = O(1)\sum_{n=1}^{m} \varphi_{n}^{k-1} \biggl( \frac{p_{n}}{P_{n}} \biggr) ^{k} |\lambda_{n} |^{k-1} | \lambda_{n} |{| z_{n}|^{k}} \\ & = O(1)\sum_{n=1}^{m}\varphi_{n}^{k-1} \biggl( \frac{p_{n}}{P_{n}} \biggr) ^{k} | \lambda_{n} |{| z_{n}|^{k}}=O(1)\quad \text{as }m\rightarrow\infty, \end{aligned}$$

in view of (12), (14) and (15). Finally, the proof of Theorem 3.1 is completed.

## Corollary

If we take $\varphi_{n}=\frac{P_{n}}{p_{n}}$ and $t_{nv}=\frac {p_{v}}{P_{n}}$ in Theorem 3.1, then we get Theorem 2.1. In this case, conditions (13) and (14) reduce to conditions (4) and (5), respectively. Also, the condition ‘$(\frac{\varphi_{n}p_{n}}{P_{n}} )$ is a non-increasing sequence’ and the conditions (10)-(12) are clearly satisfied.

## Conclusions

In this study, we have generalized a well-known theorem dealing with an absolute summability method to a ${\varphi}-|T,p_{n}|_{k}$ summability method of an infinite series by using almost increasing sequences and *δ*-quasi-monotone sequences.
